# A model for solving the prescribed burn planning problem

**DOI:** 10.1186/s40064-015-1418-4

**Published:** 2015-10-21

**Authors:** Ramya Rachmawati, Melih Ozlen, Karin J. Reinke, John W. Hearne

**Affiliations:** School of Mathematical and Geospatial Sciences RMIT University, Melbourne, Australia; Mathematics Department, Faculty of Mathematics and Natural Sciences, University of Bengkulu, Bengkulu, Indonesia

**Keywords:** MIP, Prescribed burns, Fuel reduction planning, Optimisation, Heuristics, Wildfires, Fuel management

## Abstract

The increasing frequency of destructive wildfires, with a consequent loss of life and property, has led to fire and land management agencies initiating extensive fuel management programs. This involves long-term planning of fuel reduction activities such as prescribed burning or mechanical clearing. In this paper, we propose a mixed integer programming (MIP) model that determines when and where fuel reduction activities should take place. The model takes into account multiple vegetation types in the landscape, their tolerance to frequency of fire events, and keeps track of the age of each vegetation class in each treatment unit. The objective is to minimise fuel load over the planning horizon. The complexity of scheduling fuel reduction activities has led to the introduction of sophisticated mathematical optimisation methods. While these approaches can provide optimum solutions, they can be computationally expensive, particularly for fuel management planning which extends across the landscape and spans long term planning horizons. This raises the question of how much better do exact modelling approaches compare to simpler heuristic approaches in their solutions. To answer this question, the proposed model is run using an exact MIP (using commercial MIP solver) and two heuristic approaches that decompose the problem into multiple single-period sub problems. The Knapsack Problem (KP), which is the first heuristic approach, solves the single period problems, using an exact MIP approach. The second heuristic approach solves the single period sub problem using a greedy heuristic approach. The three methods are compared in term of model tractability, computational time and the objective values. The model was tested using randomised data from 711 treatment units in the Barwon-Otway district of Victoria, Australia. Solutions for the exact MIP could be obtained for up to a 15-year planning only using a standard implementation of CPLEX. Both heuristic approaches can solve significantly larger problems, involving 100-year or even longer planning horizons. Furthermore there are no substantial differences in the solutions produced by the three approaches. It is concluded that for practical purposes a heuristic method is to be preferred to the exact MIP approach.

## Background

Fire is a natural ecosystem process. However, uncontrolled wildfires can cause significant damage. Loss of human life, destruction of properties and natural resources are amongst the problems caused by wildfires (King et al. 2008). An increase in wildfire severity and extent has been observed in many countries such as the USA, Canada, Australia, and also in southern Europe (Boer et al. 2009). This is, to some extent, due to fire suppression-focused twentieth century fire management practices, which according to Loehle (2004) and Reinhardt et al. (2008) results in uncharacteristically high fuel loads.

There are three key factors affecting fire behaviour: fuel, weather, and topography. Among these factors, it is acknowledged that only fuel can be actively controlled or managed (Schmidt et al. 2008). Finney (2007), Reinhardt et al. (2008) and Kim et al. (2009) also recommend reducing fuel load as the best possible way to slow fire growth. Fuel management is the process of altering the amount and structure of fuels through methods including prescribed burning and mechanical clearing (King et al. 2008). Fuel management is undertaken for wildfire hazard reduction as well as for ecological restoration (Reinhardt et al. 2008; Penman et al. 2011). Consequently, much effort is expended by these counties in the planning, prioritising and operational activities of prescribed burning.

Fuel management is a complex activity that involves both spatial and temporal decisions (Belval et al. 2014) and handling multiple fuel and ecological objectives. The development of decision support tools for fuel management programs is an ongoing and active research area (Martell 2011). Operations research (OR) has been successfully applied to a wide range of problems related to fire management, forestry management, and ecological management (Martell 2007) and offers great value in providing land management agencies with a framework for optimising fuel reduction planning over the landscape. A discussion of OR techniques used for solving fuel management problems can be found in the review paper by Minas et al. (2012). As an example, Wei et al. (2008) formulated an integer programming approach to reduce expected loss incurred on a landscape. Wei (2012) later proposed a mixed integer programming (MIP) method to locate fuel reduction treatments to set up potential control locations for future fires. Minas et al. (2014) later developed a model that deals with fuel treatment scheduling to break the connectivity of high risk treatment units applied in a landscape. However, a limitation of the models proposed, such as that by Minas et al. (2014), is that it only handles a single vegetation type, and fuel accumulation is treated as a linear function of time. In reality, the fire landscape is made up of multiple vegetation types, of mixed ages, with fuel accumulation taking on non-linear functions depending on vegetation type. The model presented in this paper addresses these limitations by formulating a model within a landscape that consists of multiple vegetation types of mixed ages, with differing non-linear fuel accumulation functions.

More recently, a review paper written by Chung (2015) highlighted the complexity of fuel treatments and examined previous fuel treatment optimisation studies to deal with it. Of note is that few studies incorporate the spatial and temporal dimensions of the problem. Perhaps more importantly is the conclusion that “most existing optimisation models suffer from problem complexity and a computationally intensive process ... making them almost impractical for field applications” (Chung 2015, p. 50). There is a clear need to understand the fitness for purpose of our models and to move beyond proof of concept applications. Do the trade-offs warrant obtaining the perfect solution? Can we obtain a near-optimal solution with heuristic approaches? To answer these questions, we illustrate both exact and approximate methods with a series of computational experiments with a case study of the Barwon-Otway district of Victoria, Australia. We develop a MIP model for prescribed burn planning. The objective function of the model is to reduce fuel load accumulation in a landscape of multi-age, multiple vegetation types, with differing non-linear fuel accumulation functions.

The complex multi-period model proposed in this paper can be solved exactly using an MIP solver or can be decomposed into single-period sub problems. The single-period sub problems are solved exactly using a solver and approximately using a greedy heuristic. With the exact MIP approach, an optimal solution can be achieved. However, the computational effort is costly. We introduce the two heuristics because the problem is NP-hard. With the single-period heuristic approaches, less computational effort is needed, but the solution may not be optimal as the exact MIP approach. The three approaches for solving the model are compared in terms of model applicability, computational time and the objective values.

## Model formulation

In this paper, candidate locations for fuel reduction burns are represented by ‘treatment units’. A treatment unit is defined as any area of land considered suitable for a planned burn treatment. Private land and water bodies, such as rivers and lakes, are considered non-treatable areas and are excluded from the model. Within the dataset, a treatment unit is represented as a spatial feature or polygon and contains additional attributes relating to the land ownership, vegetation types, vegetation ages and geometric properties, such as size, that exist within that treatment unit. The treatable area within the treatment unit is defined as the areas that have non-zero fuel loads.

The problem addressed by the model in this paper is where and when to conduct fuel reduction by minimising the total fuel load accumulation while still considering the ecological requirements of the vegetation present. The ecological requirements can be described as the minimum and maximum Tolerable Fire Intervals (TFI). The minimum TFI is defined as the minimum time required between two consecutive fire events at a location and is normally based on the time to reach maturity of the sensitive species in the vegetation class, while the maximum TFI refers to the maximum time needed between fire events at a location that considers the fire interval required for fire-adapted species rejuvenation (Cheal 2010). A treatment unit should not be treated if the age of vegetation growing in that location is under minimum TFI. In contrast, treatment units with vegetation over the maximum TFI must be treated.

The prescribed burning planning problem in this paper is NP-hard. The Knapsack Problem (KP), a well-known NP-hard problem (Garey and Johnson 1979), can be transformed to the 1-year planning horizon Prescribed Burn Planning (PBP) problem in polynomial number of steps. The objective function of the KP is to maximise total profit, i.e. given a set of items, each with a weight and profit, determine the items to include so that the total weight is less than or equal to a given capacity limit. In order to transform KP into PBP, the capacity limit of the regular KP is changed to the burn limit. The items are transformed to the treatment units; the weight of the items is changed to the areas, and the profits become the fuel loads. The minimum and the maximum TFI of the problem are set to infinity.

We consider the landscape divided into treatment units. It is assumed that all the vegetation of each kind is of the same age within each treatment unit. With the decision to determine when and where to treat every year to minimise total fuel load of certain regions, the following MIP model is formulated.

Sets:

$$V_{i}$$
is the set of vegetation types growing in treatment unit *i*
*T*: is the planning horizon
*C*: is the set of treatment units of which total fuel load is to be minimised

Indices:

- *i* = treatment unit
- *j* = vegetation type
- *k* = vegetation age
- *t* = period, *t* = 0, 1, 2, ...

Parameters:

- $w_{i}$ = relative importance (weight) of treatment unit *i*
- $m_{i,j}$ = the age of vegetation type *j* in treatment unit *i* at the beginning of the time-period
- $A_{i,j}$ = area (in hectares) of treatment unit *i* with vegetation type *j*
- *R* = the total treatable area in a landscape
- $\rho$ = treatment level (in percentage), i.e. the maximum proportion of the total treatable area in a landscape selected for treatment
- $c_{i}$ = area of treatment unit *i* (where $c_{i}=\underset{j}{\sum }A_{i,j}$)
- $L_{j,k}$ = fuel load (ton/hectare) of vegetation *j*, at age *k*
- $maxTFI_{j}$ = maximum TFI of vegetation type *j*
- $minTFI_{j}$ = minimum TFI of vegetation type *j*

Decision variables:

$$\begin{aligned} x_{i,t}={\left\{ \begin{array}{ll} 1 &{} \text{ if } \text{ treatment } \text{ unit }\, {i} \,\text{is treated in time period}\, {t}\\ 0 &{} \text{ otherwise } \end{array}\right. } \end{aligned}$$

$$\begin{aligned} y_{i,j,k,t}={\left\{ \begin{array}{ll} 1 &{} \text{ if } \text{ in } \text{ treatment } \text{ unit }\, i, \text{ there } \text{ is } \text{ vegetation } \text{ type }\, {j}, \text{ at } \text{ age }\, {k}, \text{ in } \text{ time }\, {t}\\ 0 &{} \text{ otherwise } \end{array}\right. } \end{aligned}$$

minimise total weighted fuel load

1 $$\begin{aligned} z=\underset{t=1}{\overset{T}{\sum }}\underset{k}{\sum }\underset{j\in V_{i}}{\sum }\underset{i\in C}{\sum }w_{i}L_{j,k}A_{i,j}y_{i,j,k,t} \end{aligned}$$

subject to

2 $$\begin{aligned} y_{i,j,k,0}=1,\quad\;\forall i,j\in V_{i},\quad k\,=m_{i,j} \end{aligned}$$

3 $$\begin{aligned} y_{i,j,k+1,t+1}\ge y_{i,j,k,t}-x_{i,t},\quad \forall i,j\in V_{i},\quad k\,=1,2,\ldots ,maxTFI_{j}-1,\forall t \end{aligned}$$

4 $$\begin{aligned} y_{i,j,k,t}\le x_{i,t},\quad \forall i,j\in V_{i},\forall t,\quad \mathrm {for}\,k=maxTFI_{j} \end{aligned}$$

5 $$\begin{aligned} y_{i,j,1,t+1}\ge x_{i,t},\quad \forall i,j\in V_{i},\forall t \end{aligned}$$

6 $$\begin{aligned} \underset{k}{\sum }y_{i,j,k,t}\le 1,\quad \forall i,j\in V_{i},\forall t \end{aligned}$$

7 $$\begin{aligned} \underset{j\in V_{i}}{\sum }\underset{k<minTFI_{j}}{\sum }y_{i,j,k,t}-\mid V_{i}\mid \underset{j\in V_{i}}{\sum }\underset{k=maxTFI_{j}}{\sum }y_{i,j,k,t}\le\, \mid V_{i}\mid (1-x_{i,t}),\quad \forall i,j\in V_{i},\forall t \end{aligned}$$

8 $$\begin{aligned} \underset{i}{\sum }c_{i}x_{i,t}\le \rho R,\quad \forall t \end{aligned}$$

9 $$\begin{aligned} y_{i,j,k,t}\in \{0,1\} \end{aligned}$$

10 $$\begin{aligned} x_{i,t}\in \{0,1\} \end{aligned}$$

The objective function (1) minimises the weighted total fuel load of all vegetation at all regions throughout a planning horizon.

Constraint (2) sets the initial conditions. Based on our observation of some raw data we felt it was necessary to include the possibility that the different vegetation types might differ in their ages. However, we assume that the all vegetation of a given type within a treatment unit will be of the same age. Constraint (3) indicates that when $x_{i,t}=0$, which means fuel treatment is not conducted, the vegetation in that area will continue growing until the following period, and the age will be incremented by one.

Constraint (4) ensures that vegetation will be treated once it has reached maximum TFI. The vegetation with age 1 in the next period comes from the areas that are treated in the current period, as denoted in constraint (5). Constraint (6) ensures that in each time-period all vegetation of a specific type in each treatment unit will be of the same age. In reality, the same vegetation type within a treatment unit may have different ages resulting from wildfires that have burnt a treatment unit partially. However, we assume that there is a representative dominant age for each vegetation type in a treatment unit. Considering the possibility of multiple ages of the same vegetation type would be computationally prohibitive. Constraint (7) enforces that the vegetation under minimum TFI cannot be treated unless there is another vegetation type in the same treatment unit which is over the maximum TFI to avoid a deadlock. However, if required, this constraint can be changed to the other way, i.e. treatment units containing young treatment units cannot be treated. Here $\mid V_{i}\mid$ represents the number of different vegetation types in treatment unit *i*.

Constraint (8) specifies that the total area selected for fuel treatment each year is not more than the annual area allotted (target) for fuel treatment (in hectares). Here, the target is obtained by multiplying the treatment level and the total treatable area in a landscape. Constraints (9) and (10) ensure that the decision variables $y_{i,j,k,t}$ and $x_{i,t}$ take binary values.

The model is capable of handling multiple vegetation types and ages. Each vegetation type has different minimum and maximum TFI, and at any period each vegetation type may have a different age even within a single treatment unit. The fuel curve representing each age of certain vegetation can also be a nonlinear function.

## Solution approaches

### An exact mixed integer programming (MIP) approach

The multi-period model discussed in "Model formulation" can be solved exactly using an MIP solver. In this subsection, the model improvement is presented to enhance the solution time.

The solution time of a MIP problem can generally be improved by reducing the number of variables, or restricting the values that they can take. Age index *k* should be based on the set of possible ages that vegetation type *j* can take in treatment unit *i* at time *t*. The maximum possible periods between two consecutive treatments for any treatment unit can be derived by finding the minimum of the maximum TFI values of all vegetation types available within that unit. This sets an upper limit on the values *k* can take within that treatment unit.

We can also tighten the MIP formulation by introducing valid inequalities on the frequency of treatment event in each unit as follows

$a_{i,j}$ = initial age of vegetation type *j* at treatment unit *i*

$$q=\mathrm {min}(maxTFI_{j}-a_{i,j})$$

11 $$\begin{aligned} \underset{t=0}{\overset{q-1}{\sum }}x_{i,t}\ge 1,\,\;\forall i \end{aligned}$$

$$p=\mathrm {min}(maxTFI_{j})$$

12 $$\begin{aligned} \underset{t}{\overset{t+p-1}{\sum }}x_{i,t}\ge 1,\,\;\forall i\ for\ t=0,1,\ldots ,T-p \end{aligned}$$

13 $$\begin{aligned} x_{i,t}=0,\;\forall i,\forall t\ \mathrm {such\ that\ }t<\mathrm {min}(\mathrm {min}(minTFI_{j}-a_{i,j}),\mathrm {min}(maxTFI_{j}-a_{i,j})),\ j\in V_{i} \end{aligned}$$

Constraint (11) ensures that a treatment unit will be treated when the most critical vegetation type (i.e., the vegetation type which sets the minimum of the maximum TFI value among all vegetation types available within a treatment unit) reaches its maximum TFI. In other words, we have to treat the treatment unit at some time in the first *q* periods of the planning horizon. Constraint (12) generalises this idea to the rest of the planning horizon by setting a frequency to treat. It ensures that treatment unit *i* must be treated at least once every *p* years. It is assumed that each treatment unit has a critical vegetation type (i.e. the vegetation in the treatment unit which has the least maximum TFI) that determines the treatment cycle. However, Constraint (12) can only help to speed up the computation time when the planning horizon is longer than the burning frequency in the treatment units. Constraint (13) reduces the number of binary variables by setting the burn variables to 0 for burns that are not allowed based on the TFI values. We considered improving the solution time by treating variable $y_{i,j,k,t}$ as a continuous variable instead of a binary variable. In other words, we replace Constraint (9) with Constraint (14) as follows

14 $$\begin{aligned} 0\le y_{i,j,k,t}\le 1 \end{aligned}.$$

### Single-period heuristic approach

In this subsection, two single-period heuristic approaches: an exact method for the single-period problem and an approximate method for the single-period problem are presented. These approaches, which are a single period 0/1 knapsack problem and a basic ‘greedy’ algorithm, are conducted as follows.

Consider *I* is the set of all treatment units in the landscape. The landscape is grouped into three disjoint sets: $I_{old}$, $I_{middle}$ and $I_{young}$. The first set, $I_{old}$, is the set of treatment units where at least one of the vegetation ages are over the maximum Tolerable Fire Interval (TFI). The second set, $I_{middle}$, is the set of treatment units where the vegetation ages are between the minimum and the maximum TFI, and nothing is over maximum TFI. The third set, $I_{young}$, is the set where all vegetation ages under maximum TFI and at least one vegetation under the minimum TFI. Here, $I=I_{old}\cup I_{middle}\cup I_{young}$. Using these parameters,

$A_{i}$ is area of treatment unit *i*

*R* is the total treatable area of the landscape

$\rho$ is treatment level (in percentage),

then the value of $r=\sum \nolimits _{i\in I_{old}}{A_{i}}$ can be determined. There are two cases that may arise when comparing the values of *r* and $\rho R$.

#### Case 1: $r\ge \rho R$

If $r\ge \rho R$, then $x_{i}$ = 0, for $i\in I_{middle}\cup I_{young}$. Either of these two approaches may be applied:

##### Using an exact method for the single period problem

The next step is to run the following model, maximise (15) subject to (16), with *i* is defined only for $I_{old}$. Here, $\rho _{new}$ is the new treatment level (in percentage), where $\rho _{new}=\rho$. maximise total fuel load:

15 $$\begin{aligned} z=\underset{i}{\sum }L_{i}x_{i} \end{aligned}$$

subject to

16 $$\begin{aligned} \underset{i}{\sum }A_{i}x_{i}\le \rho _{new}R, \end{aligned}$$

where $L_{i}$ is the total fuel load of treatment unit *i* , and $x_{i}$ is a binary variable, that is

$$\begin{aligned} x_{i}={\left\{ \begin{array}{ll} 1 &{} \text{ if } \text{ treatment } \text{ unit }\, {i} \text{ is } \text{ treated }\\ 0 &{} \text{ otherwise } \end{array}\right. } \end{aligned}$$

The objective function (15) is to maximise the total fuel load of all treatment units to be treated, subject to the single constraint (16). This constraint limits the area that can be treated per year. The model will choose the treatment units containing the highest fuel load to be burned each year. Note that the objective function (15) is different from the original objective function (1). The objective of the original problem is to minimise the total fuel load that remain in the landscape. Conversely, the objective of the single-period problem is to maximise the total fuel load that can be taken from the landscape.

##### Using an approximate method for the single-period problem

The treatment units are sorted based on the highest fuel load per area of treatment unit in the landscape, hence determining the rank or priority to burn. The treatment units then are selected by this rank until the burn limit requirement, $\rho R$, is met.

"Using an exact method for the single period problem" provides an exact solution using Integer Programming and "Using an approximate method for the single-period problem" provides an approximate solution based on the exact solution of the continuous knapsack problem.

#### Case 2: $0\le r<\rho R$

If $0\le r<\rho R$, then $x_{i}$ = 1 for $i\in I_{old}$. Either of these two approaches may be applied:

##### Using an exact method for the single period problem

The next step is to maximise (15) subject to (16) with *i* is defined only for $I_{middle}$. Here, $\rho _{new}=\rho -\frac{r}{R}$.

##### Using an approximate method for the single period problem

The same process of ranking and selecting as with the Case 1 in "Using an approximate method for the single-period problem" is undertaken until the burn limit requirement, $\rho R-r$, is met.

The approximate method can fail if we cannot use the capacity fully. The performance should get better if we have many small treatment units that we can burn to use the capacity (almost) fully.

## Model demonstration

**Fig. 1:**
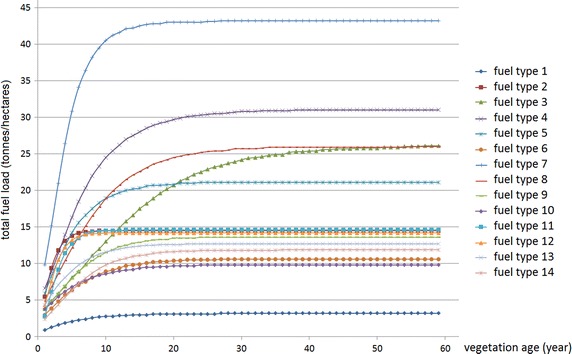
Fuel load accumulation curves over time for different fuel types listed for the Barwon-Otway region

**Fig. 2:**
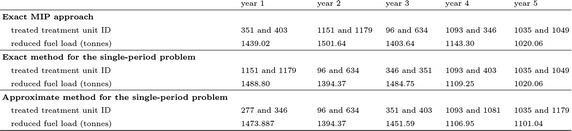
Fuel treatment outcomes, for a 5 % treatment level (40 treatment units)

**Fig. 3:**
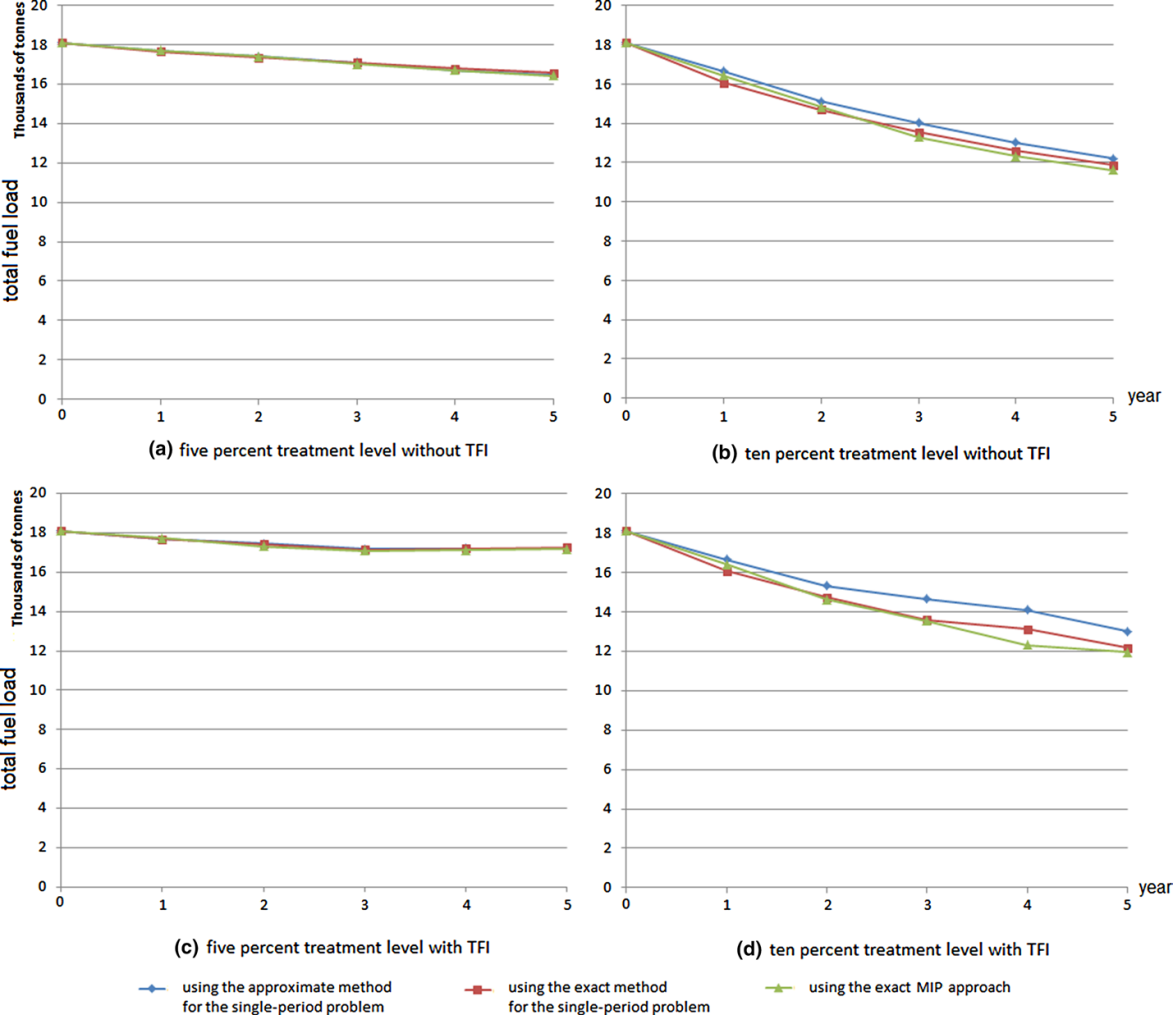
Fuel load over time for 40 treatment units with a 5-year planning horizon for the reduced study area

Consider a landscape divided into 40 treatment units. The area of each treatment unit, vegetation type and age are described in Table 1. The data regarding the minimum and the maximum TFI and the fuel type of each ecological vegetation class (EVC), can be seen in column two to five in Table 2. Figure 1 represents the fuel curve for each age of the certain vegetation type. Based on this data, some computational experiments were conducted to demonstrate three approaches: the exact MIP, the exact single-period and the approximate single-period problem. For the three approaches, we ran 5 and 10 % treatment levels, with and without TFI requirements. Figure 2 represents the fuel treatment schedule for the 5-year planning horizon with TFI requirements. The total fuel load resulting from the experiments for the 5-year planning horizon is represented in Fig. 3.

From these figures, it is clear that the 10 % treatment level results in less total fuel load than that of the 5 % treatment level. For this small landscape with the 5-year planning horizon and with TFI, the three approaches show no substantial differences, which is most likely due to the relatively small feasible region. Without TFI requirements, the feasible region will be larger than if TFI is included. This larger feasible region makes the exact MIP approach superior to the other two approaches.

We also ran experiments for ten and 15-year planning horizon with the three approaches, with and without TFI requirements. Table 3 represents the solution times and objective values for these experiments. The solution time rises as the length of the planning horizon expands. The approximate approach for the single-period problem has the lowest solution time of all, but the solution quality is also less than the other two approaches. In this small landscape, the exact approach for the single-period problem does not always outperform the approximate approach for the single-period problem, because of factors such as randomness and size of treatment units.

**Fig. 4:**
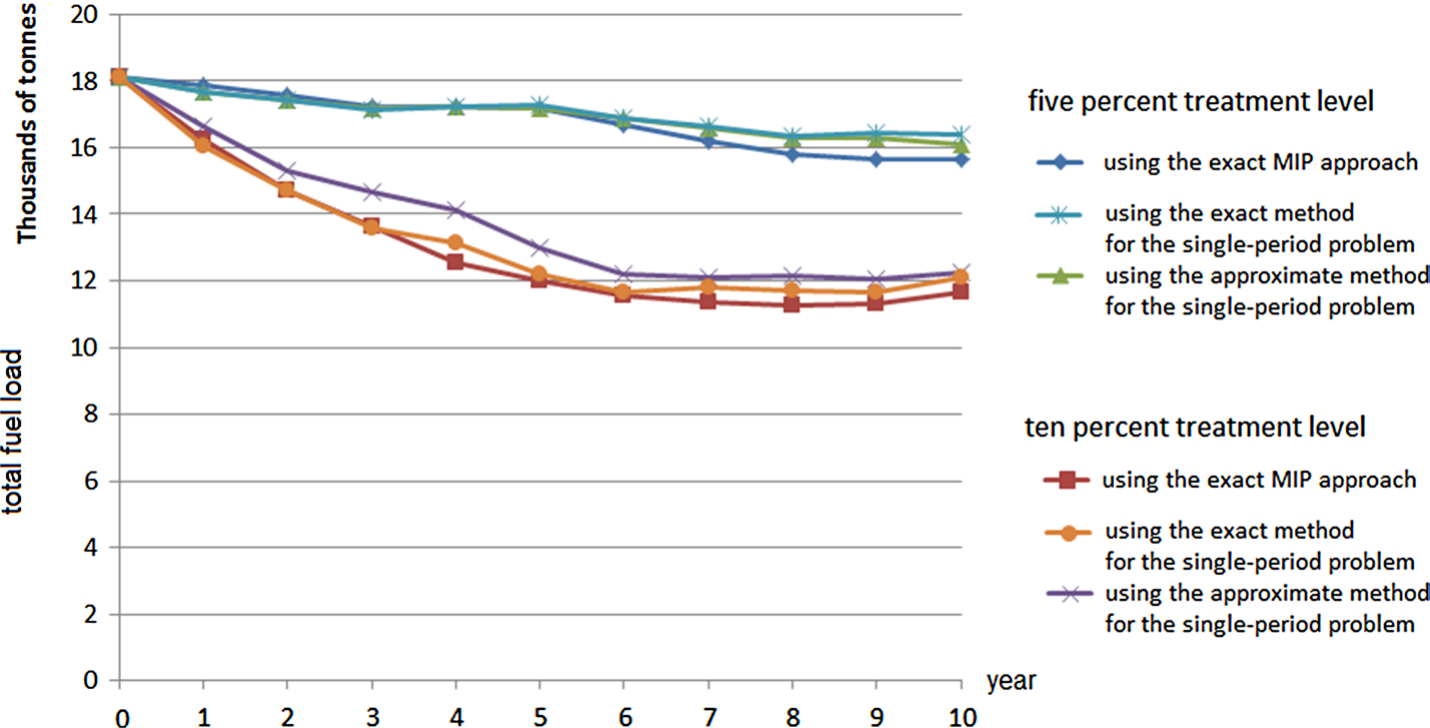
Fuel load over time for 40 treatment units with 10-year planning horizon, with TFI

The results for the 5 and 10 % treatment levels with 10-year planning horizon with TFI are described in Fig. 4. This figure shows that for each treatment level, the result of the exact MIP approach and the exact single-period problem shows no substantial difference.

Overall, in this small landscape, the result obtained by the exact method for the single-period problem is as good as that of the exact MIP approach. In "An Australian case study", the three approaches are applied in a larger landscape.

**Table 1:** 40 treatment units data containing vegetation type, extent and age

Treatment unit ID	EVC code	Area (ha)	Age (years)	Treatment unit ID	EVC code	Area (ha)	Age (years)	Treatment unit ID	EVC code	Area (ha)	Age (years)	Treatment unit ID	EVC code	Area (ha)	Age (years)
96	20	6.07	14	298	6	6.94	37	813	45	14.39	37	1093	48	13.15	37
96	164	5.52	14	306	48	26.02	37	813	21	9.30	37	1093	161	12.81	37
96	21	11.02	14	306	161	1.69	37	831	48	3.47	6	1093	163	1.15	37
96	22	1.02	14	310	48	22.49	37	831	16	24.16	6	1107	22	23.59	2
96	55	0.72	14	310	161	1.70	37	831	198	1.26	6	1107	47	1.48	2
115	71	26.23	35	346	48	8.81	38	833	48	2.89	2	1121	20	26.43	6
127	161	0.51	2	346	16	13.01	38	833	1	2.56	2	1125	20	14.15	5
127	3	24.64	2	346	198	1.36	38	833	6	1.58	2	1125	22	13.20	5
139	233	1.53	3	351	16	26.61	37	833	161	18.32	2	1130	20	27.54	14
139	45	21.79	3	351	48	0.76	37	987	16	7.29	1	1134	16	13.79	3
139	30	2.06	3	376	48	25.18	37	987	8	3.04	1	1134	20	1.60	3
169	8	6.92	5	384	175	23.86	2	987	45	16.09	1	1134	22	7.72	3
169	16	21.78	5	403	48	24.01	37	1033	45	25.74	81	1151	20	25.69	15
180	45	25.79	8	477	16	24.76	6	1035	3	2.03	37	1152	20	18.74	4
192	45	24.63	81	602	16	20.27	6	1035	161	17.48	37	1152	47	8.33	4
236	16	5.96	5	602	48	2.59	6	1035	163	5.62	37	1171	851	3.39	3
236	48	20.52	5	602	23	1.84	6	1049	16	7.97	16	1171	20	4.91	3
277	16	26.95	52	634	16	27.27	36	1049	48	18.69	37	1171	22	19.55	3
298	48	12.92	12	796	8	18.33	10	1081	48	14.97	25	1179	20	25.65	27
298	161	5.28	37	796	48	10.25	10	1081	16	6.82	25	1181	16	19.08	8
				813	16	4.03	37	1081	178	2.22	55	1181	20	7.29	8

**Table 2:** Ecological vegetation class (EVC) and associated fuel types

EVC name	EVC code	Min TFI	Max TFI	Fuel type	Area (hectare)	Area (percentage)	Initial fuel load (ton)
Creekline grassy woodland	68	20	150	7	6.14	0.008	65.08
Hills herb-rich woodland	71	15	150	7	641.42	0.872	6545.51
Creekline herb-rich woodland	164	15	150	7	281.36	0.383	2409.04
Grassy woodland	175	5	45	7	141.44	0.192	1285.21
Valley slopes dry forest	177	10	100	7	12.40	0.017	131.44
Sedgy riparian woodland	198	20	85	7	532.54	0.724	4946.06
Scoria cone woodland	894	4	15	7	20.74	0.028	219.84
Wet forest	30	45	300	9	218.10	0.297	9396.53
Shrubby wet forest	201	25	150	9	825.47	1.123	34,644.30
Riparian forest	18	10	80	10	3.56	0.005	92.29
Swampy riparian woodland	83	15	125	10	1.89	0.003	43.65
Riparian scrub or swampy riparian woodland complex	17	10	80	11	2561.76	3.484	30,299.40
Wet sands thicket	233	15	90	11	27.27	0.037	370.87
Stream bank shrubland	851	15	90	11	38.32	0.052	521.15
Cool temperate rainforest	31	45	999	1	0.60	0.001	5.88
Wet heathland	8	12	45	13	1416.63	1.926	18,692.73
Damp heath scrub	165	10	90	13	1142.88	1.554	15,908.60
Damp heath scrub/heathy woodland complex	836	10	90	13	16.05	0.022	234.33
Sand heathland	6	8	45	14	132.81	0.181	1684.73
Clay heathland	7	10	45	14	30.58	0.042	405.60
Coastal dune scrub or coastal dune grassland mosaic	1	10	90	1	253.84	0.345	3016.53
Coastal headland scrub	161	8	90	1	1077.69	1.466	12,587.77
Coastal headland scrub/Coastal tussock grassland mosaic	162	8	90	1	98.98	0.135	1177.86
Coast gully thicket	181	10	90	1	1.67	0.002	15.52
Coastal alkaline scrub	858	10	70	1	11.82	0.016	140.65
Coastal saltmarsh/mangrove shrubland mosaic	302	8	90	2	4.52	0.006	14.46
Coastal tussock grassland	163	5	40	3	260.27	0.354	3773.91
Heathy woodland	48	5	45	4	15,985.16	21.738	313,589.23
Shrubby woodland	282	10	45	4	220.56	0.300	3465.91
Lowland forest	16	8	80	5	21,454.24	29.175	574,823.49
Heathy dry forest	20	10	45	5	3958.52	5.383	95,741.43
Shrubby dry forest	21	5	45	5	2299.87	3.128	64,937.21
Grassy dry forest	22	5	45	6	2006.33	2.728	38,475.14
Herb rich foothill forest	23	8	90	6	1670.13	2.271	34,302.81
Shrubby foothill forest	45	8	90	6	12,945.85	17.605	258,807.84
Herb-rich foothill forest/shrubby foothill forest complex	178	8	90	6	2027.99	2.758	39,253.237
Damp sands herb rich woodland	3	10	90	7	270.13	0.367	2776.23
Valley grassy forest	47	10	100	7	397.99	0.541	4054.89
Plains grassy woodland	55	4	15	7	482.38	0.656	4589.66
Alluvial terraces herb-rich woodland	67	4	15	7	56.07	0.076	594.34

**Table 3:** Total fuel load and solution time (seconds) or optimality gap (%) at 10,800 s, for 40 treatment units with 5, 10 and 15-year planning horizon

	Using the exact MIP approach		Using the exact method for the single-period problem		Using the approximate method for the single-period problem	
	Treatment level		Treatment level		Treatment level	
	5 %	10 %	5 %	10 %	5 %	10 %
With TFI						
5-Year planning horizon						
Solution time	0.28 s	0.61 s	6.36 s	6.96 s	0.01 s	<0.01 s
Total fuel load (tonnes)	86,485.18	68,881.18	86,760.76	69,810.45	86,719.71	73,751.99
10-Year planning horizon						
Solution time	0.55 s	3.18 s	15.33 s	11.59 s	0.02 s	0.01 s
Total fuel load (tonnes)	167,073.99	126,230.60	169,480.60	130,989.90	168,897.70	134,543.14
15-Year planning horizon						
Solution time	8.80 s	7586.33	20.54 s	16.06 s	0.03 s	0.01 s
Total fuel load (tonnes)	245,671.97	185,310.04	250,616.10	193,569.77	250,348.40	195,460.34
Without TFI						
5-Year planning horizon						
Solution time	0.84 s	4.89 s	6.94 s	9.41 s	0.01 s	0.01 s
Total fuel load (tonnes)	85,312.245	68,445.858	85,518.07	68,752.81	85,416.47	71,016.17
10-Year planning horizon						
Solution time	307.87 s	(0.58 %)	13.46 s	18.26 s	0.02 s	0.03 s
Total fuel load (tonnes)	163,279.77	(123,346.30)	165,634.87	125,142.26	165,743.48	129,400.07
15-Year planning horizon						
Solution time	(4.59 %)	(8.82 %)	20.58 s	27.85 s	0.03 s	0.04 s
Total fuel load (tonnes)	(242,727.28)	(180,937.79)	245,443.79	180,974.32	246,804.43	186,476.47

## An Australian case study

**Fig. 5:**
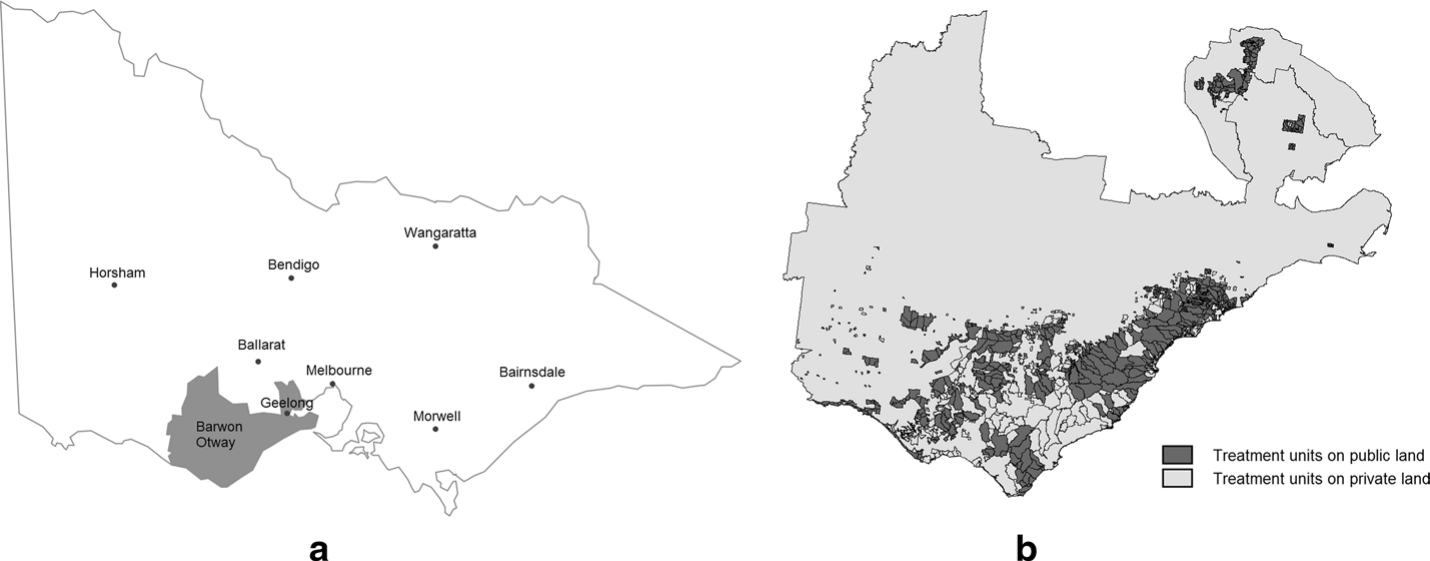
**a** Location of the case study in the Barwon-Otway district of Victoria, Australia. **b** Map showing the distribution of treatment units within the Barwon-Otway case study area

An Australian case study is presented to demonstrate the model. The study location is situated in the Barwon-Otway district of Victoria, Australia, and covers approximately 1,150,000 hectares (Fig. 5a). Data used in this case study considers land ownership, vegetation type and age in each treatment unit, minimum and maximum TFI, and fuel load for the specific age of vegetation. In this case study, we categorise the treatment units according to land ownership (i.e. public or private). It is assumed that treatments can only occur on public land, so the candidate locations for prescribed burn planning are represented in these treatment units only. A total of 711 of treatment units exist over 73,535 hectares. Figure 5b shows the public land treatment units.

Each vegetation type in this case study has its own fuel type and fuel accumulation loads over time as described in Fig. 1. The curves show that each vegetation type has a different level of fuel load depending on age. In addition, there are some aquatic vegetation types or communities that have zero fuel loads and as such require no treatment. In this paper those vegetation types are excluded. Table 2 lists the EVC name and its fuel type used in this case study.

### Using an exact mixed integer programming (MIP) approach

**Fig. 6:**
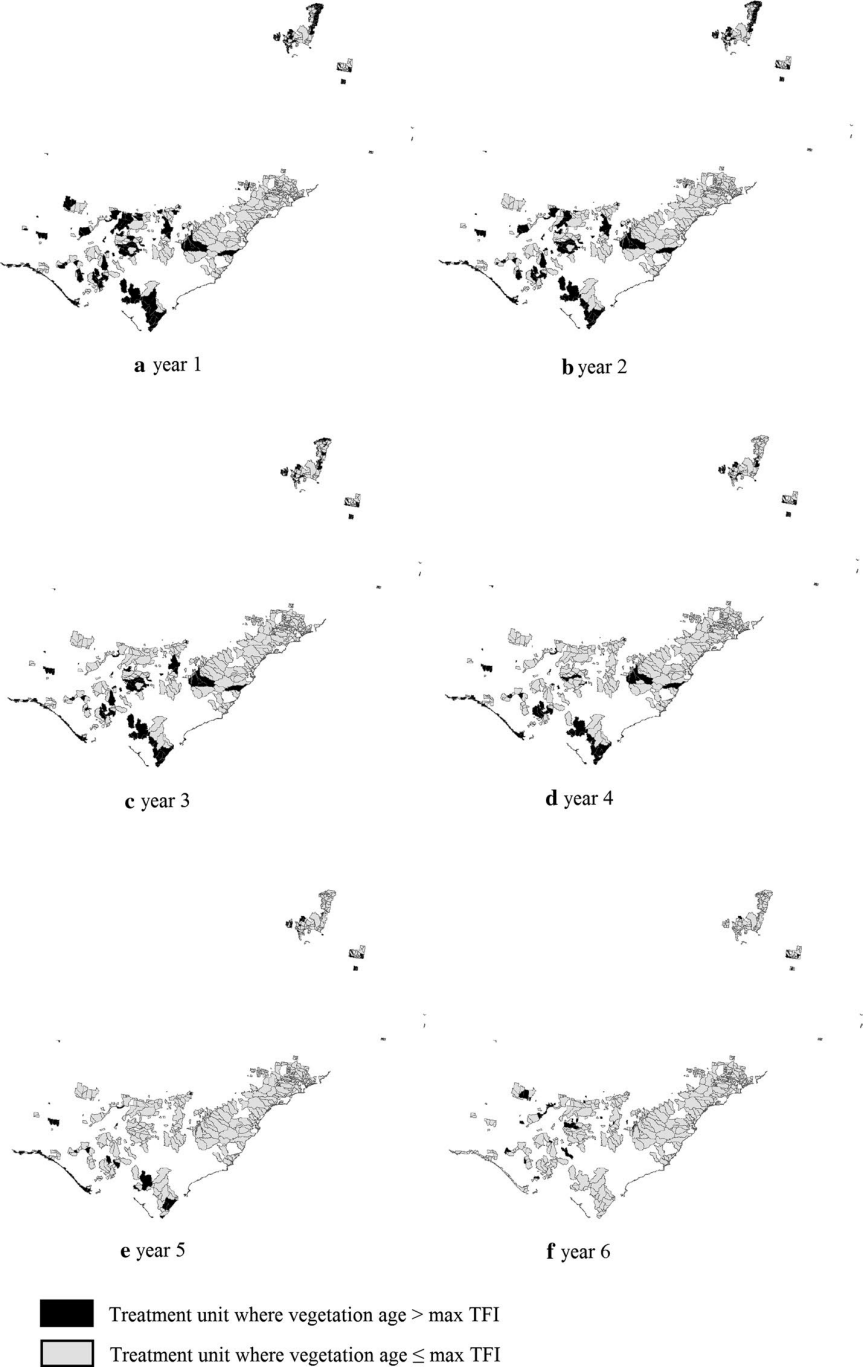
Solution of Phase 1

There are two phases when using this approach for the case study. Phase 1 (an exact method for the single-period problem) is a preliminary stage before the Phase 2 approach (an exact MIP approach) is executed. In Phase 1, the ‘old treatment units’ in the landscape are identified. The purpose of Phase 1 is to handle any infeasibility that might arise based on the initial data, by burning the old treatment units. This phase is necessary for ensuring feasibility of the Phase 2 approach. Infeasibility may arise due to conflicting constraints, especially constraints (4), (7) and (8). Constraints (4) and (7) in the Phase 2 approach require that all ‘old treatment units’ must be treated. However, treating all of these old treatment units (in this case, 35 % of the total treatable area in the landscape, as can be seen in Fig. 6a would violate Constraint (8) if the treatment level is set lower than 35 %. In practice, it would also be costly and impractical to treat such a large amount of land in a single year. Moreover, The 2009 Victorian Bushfires Royal Commission nominates a target of 5 % of the public land to be treated each year across the state in order to reduce the threat of fire for the coming fire season (Teague et al. 2010). Using a 5 % treatment level across the case study area means that imposing the maximum TFI leads to infeasibility of the Phase 2 approach. Therefore, to reduce the number of ‘old treatment units’ and achieve feasibility first, in Phase 1 the treatment level must be increased. For Phase 1 of the case study, a treatment level of 7 % of the total area of the landscape each year is imposed. Interestingly, (Penman et al. 2011) note that when more than 7 % of the total area has been burnt by prescribed fire, the total area burnt by unplanned fire will be close to zero.

Phase 1 is solved for consecutive years using the solution of the previous year as an input until the problem is reconciled, containing less than 5 % of ‘old treatment units’ in the landscape, as can be seen in Fig. 6. Based on the initial data, it would take 6 years to achieve that for our case study. The model data, now feasible, enables us to move to Phase 2.

**Table 4:** Computational comparison between the three model configurations using a 5 % treatment level

Length of planning horizon (years)	Solution time (seconds) or optimality gap (%) at 10,800 sec		
	Total	Subset	Random
5	2.12	0.47	2.02
10	43.19	6.31	37.06
15	7819.6	46.23	3194.94
20	(0.27 %)	80.79	(0.04 %)
25	(5.45 %)	265.05	(1.02 %)

**Fig. 7:**
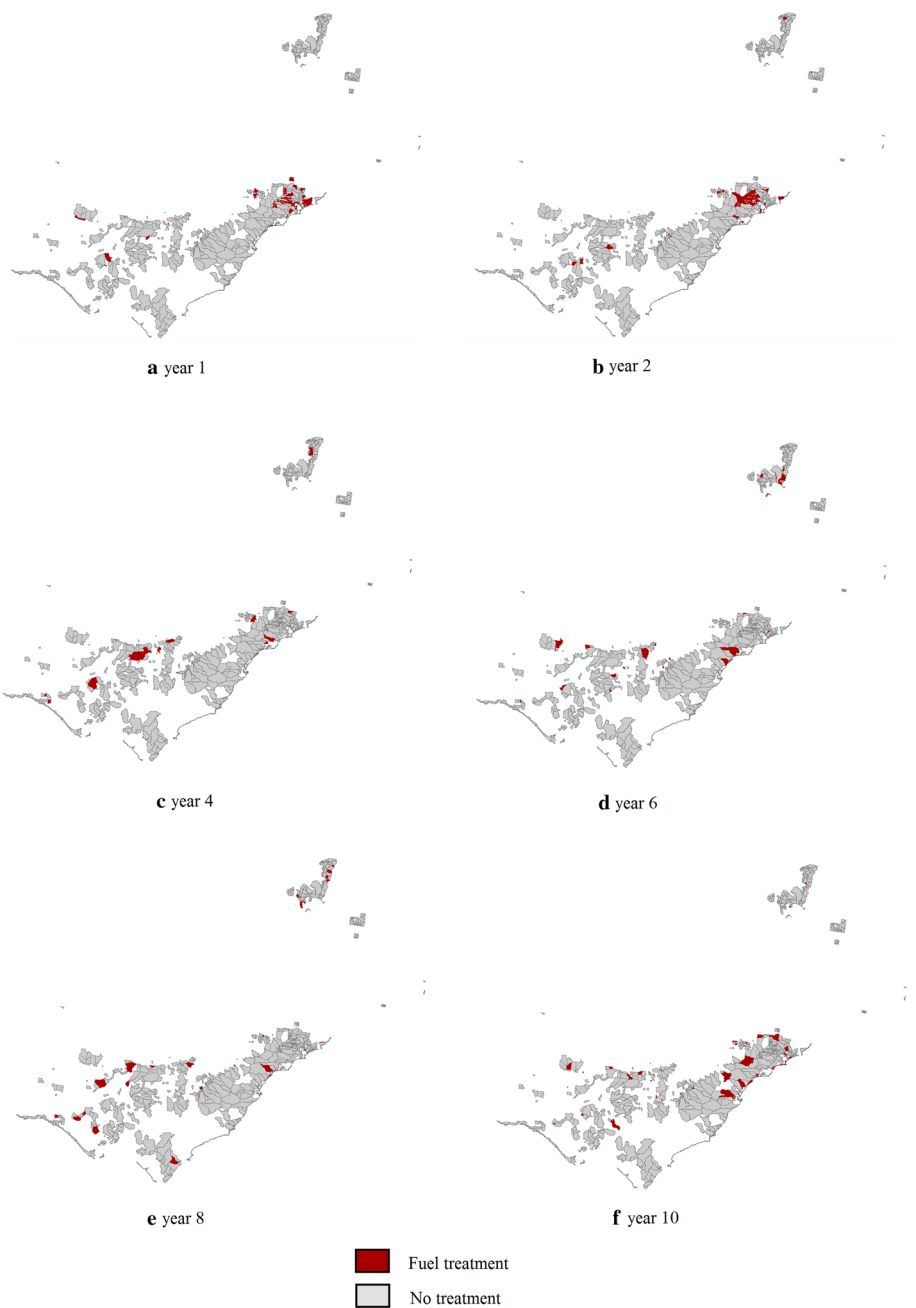
Solution of Phase 2

In Phase 2, the exact MIP approach is applied to 10-yearly planning horizons. The objective function is to minimise the total fuel load whilst meeting the constraints that have been described in "Model formulation". Figure 7 represents the result of Phase 2 and identifies the location of treatments for each year to minimise the total fuel load while satisfying the minimum and maximum TFI constraints. The length of the planning horizon is 10 years and the treatment level of each year is less than or equal to 5 %.

The model is solved using ILOG CPLEX 12.6.2 with the Python 2.7 programming language. Computational experiments are performed on Trifid, a V3 Alliance high performance computer cluster. We tested the original problem and noticed the solution time of the relaxed problem [using constraint (14)] is no better than the original most likely due to valid inequalities introduced by CPLEX. Based on that, we decided to use the original problem, not its relaxed version.

Computation time against different model configurations is tested and the results are represented in Table 4. The CPU time or the gap between the best solution identified and the current linear programming relaxation is presented. The solution may actually be optimal but CPLEX may need a long time to prove it. The three model configurations are: ‘total’ (total fuel load where all treatment units are considered equal), ‘subset’ (total fuel load where a subset of treatment units are prioritised) and ‘random’ (total fuel load where random weights are assigned to treatment units). In ‘total’, all $$w_{i}$$’s = 1. It means that the model minimises the total fuel load in all treatment units in the landscape, without prioritising certain regions. In ‘subset’, the value of $$w_{i}$$ = 1 for some priority regions, and $$w_{i}$$ = 0 for the other region. This priority may be due to proximity to towns. In ‘random’, 0 $$< w_{i} < $$ 1 assigned a relative importance weight to treatment unit which may be based on the population at risk or any other measure of defining relative importance.

**Fig. 8:**
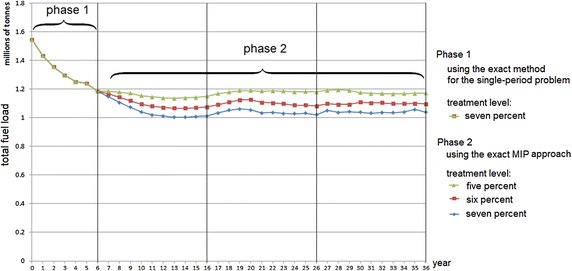
Total fuel load over time

The optimal solutions of Phase 1 and Phase 2 are represented in Figures 6 and 7, respectively. The solutions suggest where and when to conduct fuel treatments so as to minimise total fuel load accumulation. Figure 8 summarises the total fuel load over time for Phase 1 and Phase 2 for 5, 6 and 7 % annual treatment levels. From the graph it is clear that the 7 % treatment level has the least total fuel load at every point in time, which is to be expected. However, a 5 % treatment level has the most stable total fuel load in the long term. In other words, less variation is seen between years. For Phase 2 (i.e. from year 7 to 36), the approximate mean total fuel load and standard deviations in the landscape for 5, 6 and 7 % treatment level are 1.171 million tonnes (standard deviation 17,000 tonnes), 1.099 million tonnes (standard deviation 21,000 tonnes) and 1.041 million tonnes (standard deviation 29,000), respectively.

**Fig. 9:**
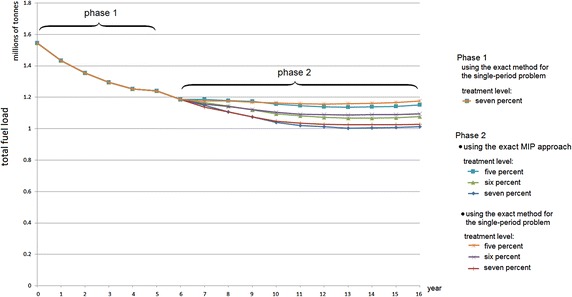
Comparison of the total fuel load using the exact MIP approach and the exact method for the single-period approach

### Using single-period heuristic approaches

Phase 2 can also be performed with the single-period heuristic approach for 5, 6 and 7 %. In Phase 2, the exact MIP approach provides a slightly better optimal solution (less total fuel load) than that of the single-period heuristic approach, as can be seen in Fig. 9. The differences between the exact MIP approach and the exact single-period heuristic approach for 5, 6 and 7 % treatment levels are 0.93, 0.94 and 1.02 %, respectively. However, using the exact MIP approach for the longer planning horizon, e.g. 100 years, is very difficult, while using the exact single-period heuristic approach a relatively good solution can be achieved in a reasonable computational time ($<$3 min for 100-year planning). Because of their practicality, in the case study we then run the model with single-period heuristic approaches for 100 years.

Both approaches (an exact method for the single-period problem and an approximate method for the single-period problem) can directly be applied to the initial data. The result from both approaches is almost identical, because there are many small treatment units in the landscape so that the burn limit requirement can be met (almost) entirely.

**Fig. 10:**
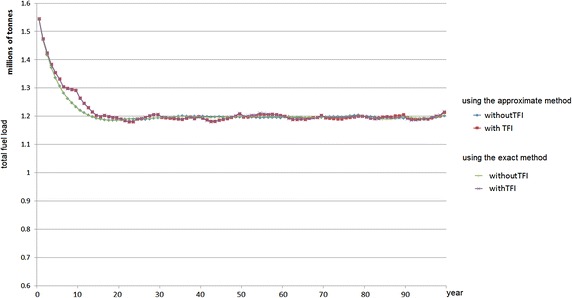
Total fuel load over time using the single-period heuristic approaches—5 % treatment level

**Fig. 11:**
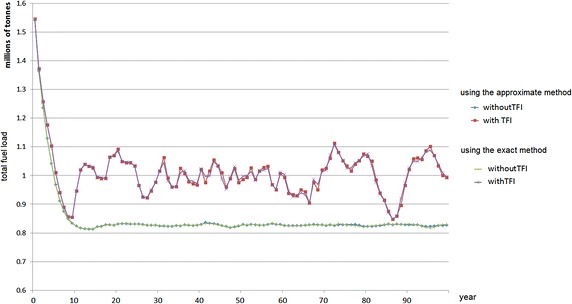
Total fuel load over time using the single-period heuristic approaches—10 % treatment level

In this case study, the computational experiments with or without incorporating TFI requirements are also conducted. The results for 5 and 10 % treatment level are represented in Figs. 10 and 11, respectively. By incorporating TFI requirements, when the treatment level is relatively high, e.g. 10 % annually, for some years the area burned may be less than 10 % in subsequent years. This is because the vegetation needs some time to regrow until it is eligible to be treated. The treatment units can only be burned if all of the vegetation types in the treatment unit are above the minimum TFI. Figures 10 and 11 also represent the results of excluding the TFI requirement, which the total fuel load in the landscape is relatively very stable. However, due to the importance of TFI as discussed in "Model formulation", in practice excluding these requirements is not recommended.

## Conclusion

The purpose of this study was twofold. Firstly, to develop an optimisation method for scheduling prescribed burns, and to embed this in a real-world case study that takes into consideration the spatial and temporal complexity of the problem. Secondly, to consider the fitness for purpose of our models by comparing the performance of simpler, heuristic-based solutions to a more complex, optimisation-based solution.

The complex multi-period model proposed takes into account multiple vegetation types of mixed ages in the landscape with differing nonlinear fuel accumulation functions. The model determines when and where to conduct fuel treatment to reduce the total fuel load in the landscape while still considering the ecological constraints relating to the Tolerable Fire Interval of each vegetation class. We compared the exact MIP and two heuristic approaches (Knapsack Problem and a greedy heuristic approach) in terms of the model tractability, computational time and the objective values.

The solution for a 15-year planning horizon for the case study comprising 711 treatment units in the Barwon-Otway district of Victoria was obtained in 2 h by using the exact MIP approach. With longer time periods it was not possible to achieve solutions of sufficient accuracy within a few days. While this approach can provide optimal solutions, it is computationally costly, especially for fuel management and ecological planning which may require longer planning horizons, and cover much larger geographic areas. Meanwhile, the heuristic methods can solve the problem for a longer times (e.g., 100 years), and the solution can be obtained in less than 3 min.

Based on our experiments, the single-period decomposition works well, and Knapsack MIP performs almost as well as the multi-period MIP. For a 10-year planning horizon with 5, 6 and 7 % treatment levels, the objective values resulting from these two approaches differs approximately by only 1 %. It is clear from the series of computational experiments that the solutions resulting from the heuristic approaches mimic that of the exact MIP to solve the prescribed burn planning model.

The results are an important reminder that future work may benefit from the use of heuristic approaches. Future research is planned to extend this study by incorporating other ecological requirements such as habitat connectivity within the landscape. However, particularly when using the exact MIP approach, this added complexity will increase computational effort to these problems as we increase the number of constraints and objectives built into our models. The case study shows that the heuristic approaches provide a near-optimal solution and the computational time is significantly faster than that of the exact MIP approach. We conclude that for practical purposes a heuristic method is more than adequate.
